# Ischemic Brain Neurodegeneration

**DOI:** 10.3390/ijms23126441

**Published:** 2022-06-09

**Authors:** Ryszard Pluta

**Affiliations:** Laboratory of Ischemic and Neurodegenerative Brain Research, Mossakowski Medical Research Institute, Polish Academy of Sciences, 02-106 Warsaw, Poland; pluta@imdik.pan.pl; Tel.: +48-22-6086-540

Aging is an inevitable phenomenon experienced by animals and humans, and its intensity varies from person to person. The result of aging is the reduction or loss of physical activity that animals and humans experience throughout their lives. With the development of modern society, people around the world are looking for a way to live longer. In recent decades, thanks to the improvement in living conditions and the changing of bad habits, people’s life expectancies have increased significantly. As a consequence, by the end of 2050, the number of people over 60 will increase to 22% [1]. Life expectancy is increasing worldwide and is expected to exceed 80 years by 2040 in most countries [2]. The hallmarks of an aging brain are metabolic and neuroglial dysregulation, impaired activity of the neural network, depletion of stem cells, loss of neuronal cells, and cognitive impairment [1,3,4]. These progressive phenomena have cognitive implications in the elderly, which may lead to accelerated neurodegeneration of the brain after stroke and Alzheimer’s disease [1,3,4]. Age-related cognitive decline is a risk factor for developing dementia, depriving older people of well-being and shortening their lives [3].

Stroke is divided into two categories: ischemic and hemorrhagic. Ischemic stroke accounts for approximately 70–85% of all cases [5,6]. Clinically, stroke is defined as local damage to the parenchyma of the brain caused by a lack of blood supply by occlusion or narrowing of an artery to a specific region, resulting in the death of neurons [5]. Ischemic stroke is a huge and growing health challenge in the modern world [7]. In developing and developed countries, post-ischemic brain neurodegeneration is common due to the progressive aging of the population. Ischemic brain neurodegeneration develops cognitive impairment in 70% of the population, is the third leading cause of disability, the second leading cause of dementia worldwide, and may soon become the leading cause of dementia [8,9]. Eighty-four percent of stroke patients in developing countries die within three years, compared to just 16% in developed countries [10]. Of the population of one million a year, 2400 will suffer a stroke, and a year later about 50% will be independent [9,10]. It is currently estimated that the number of post-ischemic patients worldwide is around 33 million with 6 million deaths, and another 5 million remain permanently disabled, putting a heavy economic burden on the family and society [5,7,8,9,11]. According to the forecast, the number of patients after ischemic stroke in the world will increase to 77 million in 2030 [9,11]. In 2010, the annual cost of treating and caring for post-ischemic patients in Europe was around EUR 64 billion [8]. In the UK, the social cost of treating stroke and loss of productivity is estimated at around £8.9 billion per year, with total care costs accounting for around 5% of the National Health System’s budget [12]. Post-stroke neurological deficits are not the main problem, the problem is a gradual, progressive decline in cognitive function resulting in increased care for these patients, and this is becoming a serious problem. In people who have had a stroke, the prevalence of dementia after the first stroke is estimated at about 10% and about 41% after the next stroke [8,9]. In long-term studies of post-ischemic dementia, its incidence has been estimated to be approximately 48% over the 25 years of follow-up [8,11]. When this trend of stroke continues, about 12 million patients will die by 2030, 70 million will survive stroke, and the world will experience over 200 million years of life with a disability each year [8,11].

Cerebral ischemia is the most common form of neurodegeneration, with a series of pathological processes that mainly occur after ischemia and gradually spread to different brain structures. Post-ischemic genetic (Table 1) and proteomic changes lead to the death of neurons in an amyloid and tau protein-dependent manner, progressive inflammation leads to brain atrophy with the development of full-blown Alzheimer’s disease-type dementia [13]. Research indicates that following ischemia, the brain may develop the neurodegeneration characteristic of Alzheimer’s disease [8,13,14].

Both human and animal stroke is a life-threatening pathological event and leads to the development of dementia with the Alzheimer’s disease phenotype [13]. First, both ischemic stroke and Alzheimer’s disease have identical risk factors. Second, the post-ischemic brain causes the death of neurons in the hippocampus, with the development of a general brain atrophy identical to that of Alzheimer’s disease [8,13,15]. Third, the neuroinflammatory response plays an important role in the progression of post-ischemic neurodegeneration of the brain as well as in Alzheimer’s disease [14]. Fourth, data suggest that post-ischemic brain neurodegeneration induces the neuropathology of Alzheimer’s disease-specific folding proteins, such as amyloid and tau protein, leading to the development of plaques, neurofibrillary tangles and cerebral amyloid angiopathy [6,13]. Fifth, dysfunction of the autophagy, mitophagy, and apoptosis (Table 2) genes is involved in post-ischemic neurodegeneration as in Alzheimer’s disease [7,13]. Sixth, data show that cerebral ischemia is a causative factor in Alzheimer’s disease [8,13,15]. The signaling phenomena generated by amyloid and tau protein post-ischemia have been argued to play a pivotal role in the development of irreversible neurodegeneration of the brain [13,15]. Thus, it has been suggested that Alzheimer’s disease-related proteins, such as amyloid and tau protein, and their genes, play a fundamental role in post-ischemic neuronal cell death and neurodegeneration.

The work of Tarkowska et al. [16] suggests that the expression of *presenilin 1* and *2* genes with age in neonatal lymphocytes after hypoxia-ischemia may be a potential biomarker to determine the severity of hypoxic-ischemia neurodegeneration and to obtain information about the time of gene changes, which may be useful for anticipating the adverse consequences of a hypoxia-ischemia episode later in life. These are the first-world data on the role of *presenilin 1* and *2* genes associated with Alzheimer’s disease in the dysregulation of neonatal lymphocytes after perinatal hypoxia. Other studies have found that high or chronic methionine intake can cause memory loss with the Alzheimer’s disease phenotype [17]. The next paper presents the joint migration of neurons through ischemic neurodegeneration of the brain and in Alzheimer’s disease [18]. It is important to thoroughly understand the role of neuronal death in neurodegeneration and identify natural or unnatural substances that can prevent their death. Advances in understanding new post-ischemic processes such as genotype (Table 1 and Table 2) and phenotype changes of Alzheimer’s disease type that have not yet been fully elucidated may facilitate the development of strategies for the prevention and treatment of post-ischemic neurodegeneration.

As life expectancy in the world increases as a result of better access and better healthcare, the number of dementia patients is increasing. Currently, Alzheimer’s disease is responsible for the largest number of dementia patients, about 60–80% of cases, followed by post-stroke dementia [19]. There are currently 50 million patients with Alzheimer’s disease worldwide, and its incidence doubles every 5 years after the age of 65 [19]. So far, two causes of Alzheimer’s disease have been intensively studied: amyloid plaques and neurofibrillary tangles, which, after more than a hundred years of research, did not explain the etiology of the disease. This was ultimately confirmed by research into anti-amyloid drugs, which showed that amyloid also has several beneficial properties, such as antibacterial, antioxidant, and neurotrophic effects [20,21,22]. In addition, the complete removal of amyloid can cause swelling and bleeding in the brain [22]. It should be noted that the causes of the disease should be investigated by looking at the body as a whole, and not just on one organ, that is, the brain. Growing evidence points to the effect of changes in the gut microbiota on slow alterations in the brain and the subsequent development of Alzheimer’s disease symptoms [22,23]. Over the course of life, inflammation in the body also translates into the development of ischemic stroke and Alzheimer’s disease [15,22,23]. 

New research on the gut microflora has shown that the microflora involved in the gut-brain axis can trigger stroke and influence post-stroke complications and treatment outcomes, and vice versa [15]. Changes in the diversity, abundance, and functioning of the gut microbiome, especially with age, known as intestinal dysbiosis, cause dysregulation in both directions of the gut-brain axis, causing changes in the gut barrier, and endotoxemia, systemic inflammation, and infections, contributing to post-stroke complications [15]. The gut-brain axis is bidirectional, and signals from the gut to the brain are passed through microbial metabolites such as amyloid, glutamate, acetylcholine, dopamine, gamma-aminobutyric acid, and serotonin, histamine, and 5-hydroxytryptamine [15,23,24]. Interestingly, several studies have shown a direct influence of intestinal dysbiosis on the development of clinical risk factors for stroke and Alzheimer’s disease, i.e., metabolic syndromes, aging, vascular dysfunction, inflammation, and leaky gut. For this reason, the gut microbiome has recently been introduced as a new important “organ” of the human and animal body [15,23], as it can affect the early neuronal and immune stages, as well as metabolic abnormalities and various pathologies [4]. The influence on the intestinal microflora may be a new direction in research on neurodegeneration after ischemia and Alzheimer’s disease, and in the treatment of both diseases suggests that we must first focus on elucidating the etiology of Alzheimer’s disease while seeking simultaneously effective nutritional or pharmacological therapies [22]. These phenomena contribute to the death of neurons, loss of synapses, and glial dysfunction in the course of stroke and Alzheimer’s disease [8,13,19,22]. Inflammatory signals from the ischemic site further enhance intestinal dysbiosis, thus causing a pro-inflammatory loop. Breaking the vicious cycle of proinflammatory phenomenon arising from the gut microbiota should be considered a promising goal in the prevention and treatment of stroke. It is already known that the harmful effects of intestinal dysbiosis can be prevented by pharmacological and non-pharmacological methods such as dietary interventions, antibiotics, prebiotics, probiotics, and synbiotics. Several studies have confirmed that the dynamics of the gut microbiome can be adjusted towards a healthy state by restoring a favorable microbial population and elevated butyric acid levels. In stroke cases, supplements rich in short-chain fatty acid-producing bacteria have been shown to significantly reduce gut leakage and inflammation; pathogenic bacteria (e.g., *Bacteroides*, *Klebsiella,* and *Haemophilus*) should be reduced, and increase the population of beneficial bacteria (e.g., *Lactobacillus, Butyricicoccus,* and *Meganonas*), which in turn inhibit neuronal cell apoptosis, oxidative stress and lead to the prevention of neurobehavioral abnormalities [5,15,19]. In summary, short-chain fatty acid producing bacteria and butyric acid supplementation have proven to be an effective treatment for stroke, although additional large-scale randomized clinical trials are needed in the future to demonstrate the efficacy and safety of short-chain fatty acids in treating patients with complications after stroke [5,15,19]. It is essential to identify conservative and reliable biomarkers of the gut microbiome to assist in the development of next-generation microbiome-based therapeutic agents for the prevention of stroke and post-stroke neurobehavioral deficits or dementia, as well as monitoring disease progression and therapeutic efficacy. Only long-term human clinical trials will help understand the effect of microbial drugs on stroke by the gut-brain axis [5].

After a stroke, there is a significant stimulation of the immune system, which, depending on the time of actions, may have a beneficial or detrimental effect on the results of ischemia. Immunity and inflammation play a key role in the pathogenesis of stroke, and the immune system is considered a promising target for limiting the progression of brain damage during or after stroke. Additionally, a special issue of “Ischemic Brain Neurodegeneration” has shown that separate subsets of monocytes may be associated with complications in the acute and subacute phases of ischemic stroke [25]. Thus, indirect monocytes may be involved in brain tissue damage in progressive infarction, while non-classical monocytes appear to be associated with infectious complications after stroke [25]. Therefore, the development of “immunomodulatory strategies” that specifically modulate the function of selected subsets of post-ischemic monocytes may be a promising therapeutic strategy [25].

There are currently no therapies to prevent the progressive changes caused by cerebral ischemia and to delay or halt post-ischemic neurodegeneration. In the absence of translational experimental post-ischemic therapies in animals for clinical use, the focus should be primarily on reducing the neurotoxic effects of amyloid and tau protein on post-ischemic neurons in order to prevent brain neurodegeneration with an Alzheimer’s disease phenotype dementia. There is a growing awareness and acknowledgment of the challenges facing countries as the number of people living with symptoms of dementia continues to increase. As an example, it is estimated that in Poland the total number of people living with cognitive deficits will more than double from 525,084 in 2018 to 1,075,099 in 2050. So people with dementia will make up 3.23% of the population in 2050 compared to 1.38% in 2018 [21]. Despite the vast amount of research investigating the causes and mechanisms leading to dementia, and projects to seek dementia-modifying or preventive therapies, there is still a lack of breakthroughs that will have a decisive impact on post-ischemic neurodegeneration and the development of Alzheimer’s disease. Moreover, the numerous failures of dementia-modifying drugs are not optimistic. Therefore, a definitive understanding of the underlying mechanisms is vitally needed to develop new therapeutic approaches. Cerebral ischemic injury and Alzheimer’s disease lead to the death of neuronal cells, which further reduces the number of neurons and reduces the neural network, negatively affecting the elderly population already with a reduced number of neurons associated with aging. In the absence of a definitive cure for cerebral ischemia and Alzheimer’s disease, it is advisable to carefully research, compare, and look for similarities in the triggering and mechanisms involved in both neuropathologies. Especially when new data show that ischemic processes may be involved in the development of Alzheimer’s disease and that there is a similarity of molecular processes between ischemic neurodegeneration of the brain and Alzheimer’s disease [13].

More research is needed to clarify whether bacterial-derived amyloid is involved in the induction and/or progression of ischemic brain neurodegeneration and Alzheimer’s disease [15,23]. However, more robust experimental evidence is still needed to show that changes in the gut microflora may be responsible for the behavioral abnormalities. Effect of microflora metabolites such as amyloid on amyloid production and accumulation, tau protein dysfunction, neuroinflammation, neuronal death, and cerebral amyloid angiopathy in various animal models, so that the crosstalk between the gut microbiome and its metabolites, and neurodegeneration in Alzheimer’s disease and post-stroke were fully understood. With the rapid advancement of research in this field, the future boom in super mechanisms and treatment of brain ischemic neurodegeneration and Alzheimer’s disease could successfully focus on research into the gut microbiome [4,5,15,22,23,24].

## Figures and Tables

**Table 1:** Changes in the expression of *amyloid protein precursor (APP), β-secretase (BACE1), presenilin 1 (PSEN1), presenilin 2 (PSEN2), and tau protein (MAPT)* genes in the CA1 and CA3 areas of the hippocampus at different times after brain ischemia.

	Genes	*APP*	*BACE1*	*PSEN1*	*PSEN2*	*MAPT*
Survival						
**CA1 region**						
**2 days**		↓	↑↑	↑	↑↑	↑↑
**7 days**		↑	↑	↑	↑	↓
**30 days**		↑	↓	↓	↓	↓
**CA3 region**						
**2 days**		↔	↓	↑	↔	↔
**7 days**		↑	↓	↑	↓	↑
**30 days**		↔	↑	↔	↑	↑

Expression: ↑↑ increase; ↑ increase; ↓ decrease; ↔ oscillation around control values.

**Table 2:** Changes in the expression of *autophagy (BECN1), mitophagy (BNIP3)*, and *apoptosis (CASP3)* genes in the CA1 and CA3 areas of the hippocampus at different times after brain ischemia.

	Genes	*BECN1*	*BNIP3*	*CASP3*
Survival				
**CA1 region**				
**2 days**		↔	↑	↑↑↑
**7 days**		↔	↔	↔
**30 days**		↔	↔	↔
**CA3 region**				
**2 days**		↔	↓	↓
**7 days**		↓	↓	↑
**30 days**		↑	↓	↑

Expression: ↑↑↑ increase; ↑ increase; ↓ decrease; ↔ oscillation around control values.

## Data Availability

The data presented in this study are available on request from the corresponding author.
